# Assessment of Body Mass Index, Polygenic Risk Score, and Development of Colorectal Cancer

**DOI:** 10.1001/jamanetworkopen.2022.48447

**Published:** 2022-12-22

**Authors:** Xuechen Chen, Hengjing Li, Marko Mandic, Michael Hoffmeister, Hermann Brenner

**Affiliations:** 1 Division of Clinical Epidemiology and Aging Research, German Cancer Research Center, Heidelberg, Germany; 2 Medical Faculty Heidelberg, Heidelberg University, Heidelberg, Germany; 3 German Cancer Consortium, German Cancer Research Center, Heidelberg, Germany; 4 Division of Preventive Oncology, German Cancer Research Center and National Center for Tumor Diseases, Heidelberg, Germany

## Abstract

**Importance:**

Excess weight, the prevalence of which is high and increasing in many countries, is linked to multiple adverse health outcomes, including increased colorectal cancer (CRC) risk. Better communication of health risks associated with excess weight might support efforts of prevention.

**Objective:**

To evaluate the individual and joint associations of body mass index (BMI) and polygenic risk with CRC, to assess potential interactions among them, and to quantify by how much increased polygenic risk for CRC can be offset by having a BMI within reference range.

**Design, Setting, and Participants:**

This population-based case-control study was conducted in the Rhine-Neckar region of southwest Germany, with recruitment from 2003 to 2017. Participants with both risk factor and genetic information were included for analysis. Data analysis was conducted from December 8, 2021, to February 17, 2022.

**Exposures:**

BMI was calculated as self-reported weight in kilograms approximately 10 years before diagnosis or interview and current height in meters squared. A polygenic risk score (PRS) was built based on 140 CRC-related risk loci.

**Main Outcomes and Measures:**

Individual and joint associations of BMI and PRS with CRC were estimated using multiple logistic regression. Associations of excess weight with CRC were quantified by adjusted odds ratios (aORs) and genetic risk equivalents (GREs), the equivalent outcomes conveyed by defined differences in PRS percentiles.

**Results:**

Among 9169 participants (median [IQR] age, 69 [62-76] years; 5589 [61.0%] male participants) included, 5053 had CRC and 4116 did not. BMI of 30 or greater was associated with higher odds of having CRC compared with BMI less than 25 (aOR, 1.71; 95% CI, 1.49-1.97), independent of PRS levels (*P* for interaction = .45). Participants with BMI of 30 or greater and a PRS in the highest tertile had higher odds of CRC compared with participants with BMI less than 25 and a PRS in the lowest tertile (aOR, 3.82; 95% CI, 3.03-4.82). The estimated association of BMI greater than 30 with CRC risk was equivalent to that of having a 41 (95% CI, 29-53)–percentile higher PRS. BMI of 30 or greater was particularly associated with stage IV CRC (aOR, 2.21; 95% CI, 1.71-2.84).

**Conclusions and Relevance:**

These findings suggest that excess weight was associated with CRC regardless of PRS levels. The association of having a BMI within reference range may be similar to that of having a substantially lower polygenic risk for CRC.

## Key Points

**Question:**

Do associations of excess weight with development of colorectal cancer differ by polygenic risk for colorectal cancer (CRC)?

**Findings:**

In this case-control study including 9169 participants (5053 CRC cases, 4116 controls), associations of excess weight with risk of CRC were independent of polygenic risk for CRC. The association of obesity with CRC risk was equivalent to that of having a 41-percentiles higher polygenic risk score.

**Meaning:**

The findings of this study could contribute to enhanced quantification and communication of the association of excess weight with CRC.

## Teaser

This case-control study assesses the individual and joint associations of body mass index and polygenic risk scores with colorectal cancer and potential interactions among them.

## Introduction

Accumulated evidence has shown that a high body mass index (BMI; calculated as weight in kilograms divided by height in meters squared), as a proxy for excess body weight, is associated with increased colorectal cancer (CRC) risk.^1,2^ It has been estimated that the global prevalence of obesity (ie, BMI ≥30), will reach 18% in men and surpass 21% in women by 2025 if post-2000 trends continue.^3^ Recent studies aimed to elucidate the mechanisms underlying the association between excess weight and CRC risk, such as obesity-related gut microbiome changes, to assess the causality of the association using Mendelian randomization approaches, and to consider more comprehensive measures of exposure, such as lifetime exposure to excess body weight.^4,5,6^ Revealing the complex interplay of overweight and obesity with genetic risk factors is also an essential aspect that might help understand the mechanisms through which excess weight promotes carcinogenesis and provide guidance for identifying subgroups who might benefit most from targeted weight intervention strategies. Polygenic risk scores (PRSs), the combination of multiple single nucleotide variations (SNVs; formerly single nucleotide polymorphisms) identified in genome-wide association studies, are increasingly used for CRC risk stratification^7,8^ and also are useful to improve limited statistical power in gene-environmental studies that often suffer from weak effects of single risk loci and harsh penalty of multiple comparison corrections.^9^ Another important issue is that CRC risks associated with excess weight and genetic risk factors are often evaluated separately, and how to communicate CRC risk increased by excess weight compared with the risk associated with background genetic profiles remains to be solved. Such comparisons might help enhance risk communication in cancer prevention, which is difficult to achieve with traditional epidemiological metrics. Therefore, the objective of this study was to explore whether the associations of BMI with CRC vary by PRS levels based on 140 previously identified variants associated with CRC risk,^8^ and to directly compare the estimated association of excess weight with the estimated association of the polygenic risk in CRC risk using a recently developed approach of genetic risk equivalent (GRE)^10,11^ to enhance communication of CRC risk associated with weight.

## Methods

### Study Design and Study Population

This case-control study was based on the Darmkrebs: Chancen der Verhütung durch Screening (DACHS), or CRC: Chances for Prevention Through Screening, study, an ongoing, large study on CRC in Germany since 2003, which has been described in detail elsewhere^12,13^ and also in the eAppendix in Supplement 1. The study population for this analysis includes cases and controls recruited between 2003 and 2017 for whom genetic data were available. The DACHS study was approved by the ethics committee of the Heidelberg Medical Faculty of Heidelberg University and the state medical boards of Baden-Wuerttemberg and Rhineland-Palatinate. Written informed consent was obtained from each participant. This study followed the Strengthening the Reporting of Observational Studies in Epidemiology (STROBE) reporting guideline for case-control studies.

### Assessment of Body Fat

Participants reported their weight at various ages (10-year intervals: eg, at ages 20, 30, and 40 years) and current weight and height during the interview. For this analysis, BMI was calculated by dividing weight in kilograms approximately 10 years preceding the year of diagnosis or interview by square of height in meters. For example, weight at age 50 years was used for participants aged 55 to 64 years, and weight at age 60 years was used for participants aged 65 to 74 years. This measure of body weight was preferred over current weight in our analyses to prevent potential bias resulting from prediagnostic weight loss caused by CRC. Underweight (ie, BMI <18.5) was extremely rare at approximately 10 years before diagnosis or interview (cases: 0.8%; controls: 0.7%), thus it was not considered as separate category in the main analyses. Therefore, BMI was categorized into 3 categories: less than 25 (normal weight), 25 to less than 30 (overweight), and 30 or greater (obesity).

### Derivation of the PRS

Details about genotyping and imputation of missing genotypes are shown in eTable 1 in Supplement 1. The PRS, integrating information from 140 CRC–risk related variants (eTable 2 in Supplement 1) that were identified in a recent genome-wide association study,^8^ was calculated by summing the number of risk alleles of the respective variants (0, 1, or 2 copies of the risk allele for genotyped loci; imputed dosages for imputed loci).

### Statistical Analysis

Distribution of baseline characteristics was assessed by descriptive statistics and compared between case and control groups using χ^2^ tests for categorical data. We also comprehensively described and compared the distribution of BMI levels at each decennial age between cases and controls, and the distribution of BMI and PRS levels between those with and without previous colonoscopy, an important factor associated with risk of CRC, among case and control groups. Additionally, we assessed the association between the PRS and BMI among controls.

Associations of BMI with CRC were assessed using logistic regression models. Model 1 was adjusted for age (at diagnosis or interview) and sex. Additional covariates adjusted for in model 2 included school education, smoking status, alcohol consumption (above vs below the recommended maximum limits of alcohol consumption: 12 g ethanol daily for women; 24 g ethanol daily for men^14^), lifetime mean physical activity, red and processed meat intake in the previous 12 months before enrollment, history of colonoscopy, history of diabetes, family history of CRC, current use of statins at least 1 time per week, regular use of nonsteroidal anti-inflammatory drugs (including aspirin ≥2 times per week for more than 1 year), and the PRS (continuous variable). We tested for multicollinearity and did not find any problematic variables (variance inflation factors of all covariates <2).

We further investigated the variations of associations between BMI and odds of CRC by PRS levels (low, medium, and high, categorized according to the tertiles of PRS among controls) and explored the joint associations of BMI and PRS categories with CRC by using participants with low PRS and normal weight as the reference group. We tested the interactions between PRS (continuous or categorical variable) and BMI levels (categorical or continuous variable) in CRC odds by adding a multiplicative term of PRS and BMI in regression models.

Accounting for potential variations by cancer sites and stage, we performed subtype analyses with different outcomes (ie, colon and rectum cancer, stage I-III CRC, and stage IV CRC).^15^ Subgroup analyses by age (≤55 years vs >55 years), sex (female vs male), history of colonoscopy (yes vs no), and family history of CRC (yes vs no) were also conducted. Interactions were tested for statistical significance by including a cross-product term of BMI categories and pertinent stratification risk factors in models.

Details of derivation and application of the GRE have been published elsewhere^10,11^ and are described specifically for this study in the eAppendix in Supplement 1. Here, GREs for different BMI categories were calculated as ratios of coefficients for BMI and PRS percentiles from logistic regression models. Interpretation of GREs is fairly straightforward. For example, a GRE of 30 for BMI of 30 or greater indicates that the increase in odds of CRC associated with obesity corresponds to having a 30-percentile higher PRS level.

All analyses were performed using R software version 4.1.1 (R Project for Statistical Computing) and SAS software version 9.4 (SAS Institute). Statistical tests were 2-sided with an α = .05. Data analysis was conducted from December 8, 2021, to February 17, 2022.

## Results

### Baseline Characteristics of Study Population

After excluding participants with missing values of BMI, 9169 participants (median [IQR] age, 69 [62-76] years; 5589 [61.0%] male participants) were included in this analysis, with 5053 participants with CRC (cases) and 4116 controls (eFigure in Supplement 1). Generally, participants with CRC more often had a lower level of school education and a higher BMI, smoked, drank alcohol, engaged in physical activity, consumed red and processed meat, and less frequently used nonsteroidal anti-inflammatory drugs and statins than controls (Table 1). In addition, more participants with CRC reported a history of diabetes and family history of CRC, and fewer reported a history of colonoscopy before diagnosis compared with control participants. We observed a higher prevalence of obesity among participants with CRC than among controls at each decennial age (eTable 3 in Supplement 1). PRSs did not vary by history of colonoscopy within participants with CRC or controls, while there was a higher prevalence of obesity among controls with history of colonoscopy (67 participants [16.8%] aged <50 years; 350 participants [16.9%] aged ≥50 years) than those without (231 participants [14.1%]) (eTable 4 in Supplement 1). In addition, we did not find any associations between the PRS and BMI in the control group.

**Table 1.** Distribution of Characteristics in Patients With CRC and Controls

Characteristic	Participants, No. (%)		*P* value
	CRC case (n = 5053)	Control (n = 4116)	
Sex			
Female	1994 (39.5)	1586 (38.5)	NR^a^
Male	3059 (60.5)	2530 (61.5)	
Age, median (IQR), y	69 (61-76)	70 (62-76)	NR^a^
Education, y^b^			
<9	3291 (65.1)	2272 (55.2)	<.001
9-10	898 (17.8)	868 (21.1)	
>10	855 (16.9)	969 (23.5)	
BMI			
<25	1541 (30.5)	1580 (38.4)	<.001
25 to <30	2372 (46.9)	1885 (45.8)	
≥30	1140 (22.6)	651 (15.8)	
Smoking status^c^			
Never	2242 (44.4)	2082 (50.6)	<.001
Former	2025 (40.1)	1577 (38.3)	
Current	763 (15.1)	446 (10.8)	
Alcohol consumption above recommended levels^d^	1312 (26.0)	936 (22.7)	<.001
Physical activity, MET-h/wk^e^			
≤121.6	1139 (22.5)	1026 (24.9)	.004
121.7-178.4	1234 (24.4)	1025 (24.9)	
178.5-244.6	1222 (24.2)	1026 (24.9)	
>244.6	1405 (27.8)	1025 (24.9)	
Red and processed meat intake^f^			
≤1 Time per week	397 (7.9)	480 (11.7)	<.001
Multiple times per week	3019 (59.7)	2491 (60.5)	
1 Time per day	1396 (27.6)	1003 (24.4)	
>1 Time per day	227 (4.5)	128 (3.1)	
Use of NSAIDs (including aspirin)^g^	1444 (28.6)	1564 (38.0)	<.001
Use of statins^h^	869 (17.2)	922 (22.4)	<.001
History of diabetes^i^	952 (18.8)	552 (13.4)	<.001
Family history of CRC^j^	734 (14.5)	451 (11.0)	<.001
History of colonoscopy	1335 (26.4)	2480 (60.3)	<.001
Polygenic risk score^k^			
Low	1062 (21.0)	1375 (33.4)	<.001
Medium	1615 (32.0)	1371 (33.3)	
High	2376 (47.0)	1370 (33.3)	

Abbreviations: BMI, body mass index (calculated as weight in kilograms approximately 10 years before diagnosis or interview divided by height in meters squared); CRC, colorectal cancer; MET, metabolic equivalent of task; NR, not reported; NSAID, nonsteroidal anti-inflammatory drug.

^a^ *P* values were not reported for the matching factors age and sex.

^b^ Data missing for 9 cases and 7 controls.

^c^ Data missing for 23 cases and 11 controls.

^d^ Data missing for 16 cases and 17 controls.

^e^ Data missing for 53 cases and 14 controls.

^f^ Data missing for 14 cases and 14 controls.

^g^ Data missing 1 control.

^h^ Data missing for 2 cases and 8 controls.

^i^ Data missing for 8 cases and 5 controls.

^j^ Data missing for 3 cases and 3 controls.

^k^ Polygenic risk score was categorized according to tertiles of polygenic risk score (low, medium, and high) among controls.

### Associations of BMI and PRS With CRC

Both PRS and BMI were independently associated with CRC risk (*P* for interaction between PRS levels and BMI categories = .45) (Table 2). Having a PRS in the top tertile was associated with significantly increased odds of CRC compared with the lowest tertile (adjusted odds ratio [aOR], 2.27; 95% CI, 2.02-2.55). Additionally, BMI approximately 10 years before diagnosis or interview was associated with increased risk of having CRC (25 to <30: aOR, 1.31; 95% CI, 1.18-1.46; ≥30: aOR, 1.71; 95% CI, 1.49-1.97) compared with normal weight.

**Table 2.** Individual Associations of PRS and BMI With CRC

Variables	Participants, No. (%)		OR (95% CI)	
	CRC case	Control	Model 1^a^	Model 2^b^
PRS^c^				
Low	1034 (20.9)	1351 (33.4)	1 [Reference]	1 [Reference]
Medium	1582 (32.0)	1351 (33.4)	1.53 (1.37-1.71)	1.57 (1.39-1.77)
High	2323 (47.0)	1346 (33.3)	2.25 (2.03-2.50)	2.27 (2.02-2.55)
BMI^d^				
<25	1501 (30.4)	1553 (38.4)	1 [Reference]	1 [Reference]
25 to <30	2323 (47.0)	1859 (45.9)	1.34 (1.21-1.47)	1.31 (1.18-1.46)
≥30	1115 (22.6)	636 (15.7)	1.86 (1.65-2.10)	1.71 (1.49-1.97)
Per 5-unit increase^d^	NA	NA	1.34 (1.27-1.41)	1.30 (1.22-1.38)

Abbreviations: BMI, body mass index (calculated as weight in kilograms approximately 10 years before diagnosis or interview divided by height in meters squared); CRC, colorectal cancer; NA, not applicable; OR, odds ratio; PRS, polygenic risk score.

^a^ Adjusted for age and sex.

^b^ Additionally adjusted for education, smoking status, alcohol consumption, physical activity, red and processed meat intake, history of colonoscopy, history of diabetes, family history of colorectal cancer, use of statins, use of nonsteroidal anti-inflammatory drugs, PRS (continuous variable, for the analysis of BMI), and BMI (for the analysis of PRS).

^c^ PRS was categorized according to tertiles of PRS (low, medium, and high) among controls.

^d^ Interactions were tested by additionally including a cross-product term of PRS (continuous variable) with BMI in multivariable models. Categorical BMI: *P* = .45; continuous BMI: *P* = .51.

In the stratified analysis by PRS levels (Table 3), very similar associations between BMI and CRC were seen within each PRS tertile, and there was no statistically significant interaction between BMI and PRS tertiles. The BMI and PRS interaction had essentially multiplicative associations, with risk of CRC increasing with PRS level among people with BMI of 30 or greater (low PRS: aOR, 1.97; 95% CI, 1.53-2.56; medium PRS: aOR, 2.79; 95% CI, 2.19-3.56; high PRS: aOR, 3.82; 95% CI, 3.03-4.82) compared with people with normal weight and low PRS (Figure; eTable 5 in Supplement 1). Likewise, people with a high PRS had increasingly high odds of CRC with increasing BMI (<25: aOR, 2.41; 95% CI, 1.98-2.93, 25 to <30: aOR, 3.14, 95% CI, 2.60-3.79; ≥30: 3.82, 95% CI, 3.03-4.82) compared with people with a low PRS and normal weight.

**Table 3.** Association of BMI With CRC by PRS Levels

BMI^a^	Participants, No. (%)		OR (95% CI)^b^^,^^c^
	CRC case	Control	
Low PRS			
<25	304 (29.4)	534 (39.5)	1 [Reference]
25 to <30	485 (46.9)	618 (45.7)	1.33 (1.08-1.64)
≥30	245 (23.7)	199 (14.7)	1.90 (1.45-2.48)
Per 5-unit increase	NA	NA	1.30 (1.16-1.46)
Medium PRS			
<25	484 (30.6)	500 (37.0)	1 [Reference]
25 to <30	751 (47.5)	632 (46.8)	1.27 (1.05-1.53)
≥30	347 (21.9)	219 (16.2)	1.69 (1.33-2.15)
Per 5-unit increase	NA	NA	1.29 (1.17-1.44)
High PRS			
<25	713 (30.7)	519 (38.6)	1 [Reference]
25 to <30	1087 (46.8)	609 (45.2)	1.32 (1.12-1.57)
≥30	523 (22.5)	218 (16.2)	1.62 (1.30-2.02)
Per 5-unit increase	NA	NA	1.29 (1.17-1.43)

Abbreviations: BMI, body mass index (calculated as weight in kilograms approximately 10 years before diagnosis or interview divided by height in meters squared); CRC, colorectal cancer; NA, not applicable; OR, odds ratio; PRS, polygenic risk score.

^a^ PRS was categorized according to tertiles of PRS (low, medium, and high) among controls.

^b^ Adjusted for age, sex, education, smoking status, alcohol consumption, physical activity, red and processed meat intake, history of colonoscopy, history of diabetes, family history of CRC, use of statins, and use of nonsteroidal anti-inflammatory drugs.

^c^ Interactions were tested by additionally including a cross-product term of PRS (categorical variable) with BMI in multivariable models. Categorical BMI: *P* = .75; continuous BMI: *P* = .81.

**Figure.**
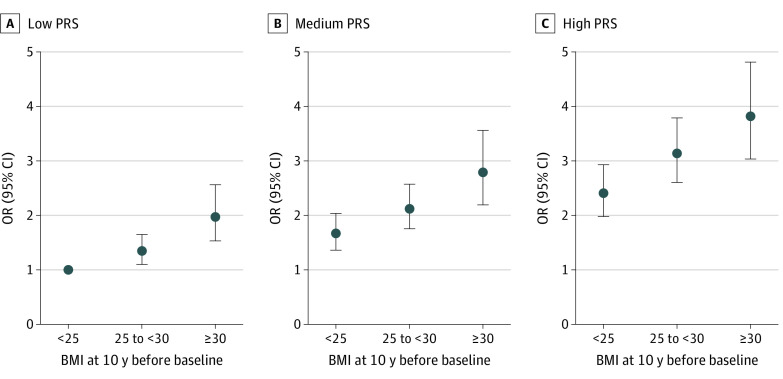
Joint Associations of Body Mass Index (BMI) Categories and Polygenic Risk Score (PRS) Levels With Colorectal Cancer PRS was categorized according to tertiles of PRS (low, medium, and high) among controls. Participants with low PRS and BMI (calculated as weight in kilograms approximately 10 years before diagnosis or interview divided by height in meters squared) less than 25 were used as the reference group. OR indicates odds ratio.

### GREs for Different BMI Categories

The estimated association of overweight and obesity with CRC risk was equivalent to the risk associated with having a 21 (95% CI, 12-30)-percentile higher PRS for participants with overweight and 41 (95% CI, 29-53)-percentile higher PRS for participants with obesity (Table 4). We did not observe variations of ORs for the associations between BMI and CRC by cancer sites, and differences in corresponding GREs were negligible. However, participants with obesity had higher risk and GREs with respect to having stage IV CRC (aOR, 2.21; 95% CI, 1.71-2.84) compared with CRC in early stage (*P* for heterogeneity = .02).

**Table 4.** Genetic Risk Equivalents by BMI

BMI	Participants, No. (%)		OR (95% CI)^a^	GRE (95% CI)
	CRC case	Control		
Overall CRC				
<25	1501 (30.4)	1553 (38.4)	1 [Reference]	0 [Reference]
25 to <30	2323 (47.0)	1859 (45.9)	1.31 (1.17-1.45)	20.6 (12.1-29.2)
≥30	1115 (22.6)	636 (15.7)	1.71 (1.49-1.96)	40.9 (29.2-52.7)
Colon cancer^b^				
<25	912 (30.3)	1553 (38.4)	1 [Reference]	0 [Reference]
25 to <30	1393 (46.3)	1859 (45.9)	1.28 (1.14-1.45)	18.8 (9.3-28.4)
≥30	703 (23.4)	636 (15.7)	1.75 (1.50-2.04)	42.7 (29.6-55.8)
Rectum cancer^b^				
<25	589 (30.5)	1553 (38.4)	1 [Reference]	0 [Reference]
25 to <30	930 (48.2)	1859 (45.9)	1.36 (1.18-1.57)	23.5 (11.8-35.2)
≥30	412 (21.3)	636 (15.7)	1.68 (1.39-2.02)	39.6 (23.9-55.3)
Stage I-III CRC^c^				
<25	1286 (30.7)	1553 (38.4)	1 [Reference]	0 [Reference]
25 to <30	1974 (47.1)	1859 (45.9)	1.28 (1.14-1.43)	18.8 (10.0-27.7)
≥30	932 (22.2)	636 (15.7)	1.63 (1.41-1.88)	37.3 (25.3-49.3)
Stage IV CRC^c^				
<25	196 (28.0)	1553 (38.4)	1 [Reference]	0 [Reference]
25 to <30	333 (47.5)	1859 (45.9)	1.57 (1.27-1.93)	34.4 (16.6-52.2)
≥30	172 (24.5)	636 (15.7)	2.21 (1.71-2.84)	60.5 (36.4-84.7)

Abbreviations: BMI, body mass index (calculated as weight in kilograms approximately 10 years before diagnosis or interview divided by height in meters squared); CRC, colorectal cancer; GRE, genetic risk equivalent; OR, odds ratio.

^a^ Adjusted for age, sex, education, smoking status, alcohol consumption, physical activity, red and processed meat intake, history of colonoscopy, history of diabetes, family history of CRC, use of statins, use of nonsteroidal anti-inflammatory drugs, and the polygenic risk score (per 10 percentiles, continuous variable).

^b^ Heterogeneity between stratum: 25 to <30: *P* = .44; ≥30: *P* = .43.

^c^ Heterogeneity between stratum: 25 to <30: *P* = .10; ≥30: *P* = .02.

Detailed GRE information for BMI categories in subgroups defined by age, sex, history of colonoscopy, and family history of CRC are presented in eTable 6 in Supplement 1. No statistically significant interactions between BMI and pertinent stratification factors were observed.

## Discussion

In this large-scale population-based case-control study with more than 5000 CRC cases and detailed ascertainment of CRC risk factors in personal interviews, we evaluated the risk of CRC according to BMI and PRS levels and also by their joint consideration. We found that the associations of BMI with CRC risk were independent of PRS levels for CRC, and observed an enhanced CRC risk stratification through joint classification by BMI and PRS. Participants with obesity had substantially higher GREs compared with persons with normal weight, underlining the benefits of having a healthy weight in CRC prevention. In addition, BMI was particularly associated with stage IV CRC.

The mechanisms of colorectal carcinogenesis associated with obesity are not fully understood. Plausible biological mechanisms include subclinical low-grade inflammation, oxidative stress, insulin resistance and abnormalities of the insulin-like growth factor 1 system, and signaling and sex hormones biosynthesis and pathways.^16^ Recent evidence also suggests a possible role of obesity-associated change of gut microbiota in the development of CRC.^4,17^ Gene-environment interaction studies might help to unravel the mechanisms through which obesity is associated with carcinogenesis. However, no firm evidence has been found after multiple testing corrections in the interaction analyses of BMI with individual CRC-related SNVs identified in genome-wide association studies.^18,19,20,21^ A 2021 study^22^ that examined the interactions between BMI and approximately 2.7 million SNVs in association with the risk of colorectal adenocarcinoma among more than 14 000 participants with colorectal adenocarcinoma and 14 000 controls found that the BMI-CRC associations were more pronounced for women with the rs4939827-CC genotype (located in the SMAD7 gene at 18q21.1) than for women with CT or TT genotypes.

Given the very small changes associated with single SNVs, the power to detect their interactions with risk factors is typically very small, in particular in light of the need for correcting for multiple testing if multiple SNVs are assessed. Statistical power is much higher for analyses of statistical interactions of PRS with environmental or lifestyle-related risk factors. To our knowledge, only 1 study from the UK published in 2020 by Yang et al^21^ evaluated interactions between a PRS and established lifestyle-related risk factors, including BMI, with respect to CRC risk. In that study, neither the main analysis of BMI nor any of the interactions between PRS and lifestyle factors were statistically significant. In our study, a clear association of BMI was seen, but the test for interaction likewise did not reach statistical significance. However, it should be noted that the same relative risk of BMI categories across PRS levels in evaluating interactions on a multiplicative scale implies a higher increase in absolute risk among individuals with higher PRS levels, due to their higher baseline risk.^23^ Thus, people with high PRS levels might benefit most from having a healthy body weight. Our findings underscore the importance of weight control, in particular for those with high susceptibility to CRC, and also provide insights to improve CRC risk discrimination through joint classification by PRS and BMI.

Another important contribution of our study is that this was the first study that quantified the association of excess weight with risk of CRC by a defined difference in PRS levels using the novel metric of GRE. The large GRE indicates that the risk associated with obesity was equivalent to that associated with having a substantially higher PRS for CRC. In other words, genetically increased CRC risk may be compensated to a substantial extent by keeping a healthy weight. This important message might be helpful for risk communication in cancer prevention efforts and therefore help to improve adherence to a healthy lifestyle, including maintaining a healthy weight.

A noteworthy result of our study is the particularly high OR and GRE with respect to stage IV CRC. The finding of a more pronounced association of BMI with advanced-stage CRC is in line with the results of a population-based cohort study with 28 098 participants from Sweden.^24^ However, other studies did not find a higher BMI among patients with stage IV CRC.^25,26,27^ One plausible explanation for this apparent inconsistency might be the different time points of BMI measurement, which was at baseline before incidence of CRC in the Swedish cohort study^24^ and approximately 10 years before CRC diagnosis in our study, whereas BMI was determined at the time of diagnosis in other studies.^25,26,27^ Unintended weight loss before cancer diagnosis may be a first sign of malignant cancer, which could explain the apparent absence of associations between obesity at diagnosis and late-stage CRC. Disparities in CRC screening^28,29^ and pronounced intertumor and intratumor heterogeneity^30,31^ might also influence the association between BMI and cancer stages. For example, previous studies^28,29^ have shown that participants with obesity were less likely to have CRC screening, which might result in delayed diagnosis and thus increase the risk of more advanced CRC. However, history of colonoscopy was adjusted for, and differences in screening history are unlikely to explain the association of obesity with advanced stage CRC in our analysis. Another plausible explanation might be that diagnosis of CRC might be delayed among people with obesity, which might lead to a higher proportion of late-stage disease at diagnosis. Further studies with careful consideration of these factors are warranted to disentangle the association of excess weight with late-stage CRC risk and to explore the potential mechanisms underlying these associations.

### Limitations

Our study has several limitations that require careful consideration. First, information bias, such as recall bias, cannot be ruled out, since most data, including self-reported weight, were gathered retrospectively through personal interviews. Second, although we adjusted for multiple relevant covariates, we cannot rule out residual confounding caused by other or imperfectly ascertained CRC- and obesity-related factors. In particular, our study lacked reasonably precise quantification of potentially important nutritional and anthropometric factors, such as fat intake, waist circumference, or duration of obesity. Third, despite the overall large size of this study, 95% CIs for ORs and the corresponding GREs were broad in some subgroups with small sample sizes, especially in the subgroup of younger adults. Fourth, it has to be noted that the PRS in this study was based on the 140 CRC-related genetic variants that have been identified so far, which only explain a proportion of genetic CRC risk. Therefore, GREs need to be interpreted with respect to currently explained rather than overall genetic risk. Fifth, our study was restricted to a European population, which limits generalizability to other populations. Sixth, our study lacked reasonable statistical power to explore the interaction of BMI with PRS in specific CRC subtypes, as tumor subtype information was available for only a proportion of cases. The individual and joint associations of excess weight and PRS with subtype-specific CRC risk should be explored in future, even larger studies.

## Conclusions

The findings of this case-control study add to the limited evidence on the individual and joint associations of BMI and PRS with CRC. The absence of interaction on a multiplicative scale between PRS and BMI, 2 significant CRC risk factors, provides a great potential for CRC risk classification through their joint consideration, and also underlines the importance of keeping a healthy weight. Although this applies to all levels of PRS, it is particularly relevant for individuals with high PRS due to the more pronounced absolute risk increase associated with overweight and obesity among them. The large GREs for obesity further underscore the role of having a normal weight, and they might help in risk communication as they illustrate that a large proportion of increased genetic risk may be compensated by adhering to the guideline for healthy weight. Further studies are warranted to provide more precise estimates of GREs in certain subgroups. This particularly applies to the younger population, among whom the incidence of CRC has been increasing in many countries in recent years.^32^

## Notes

Supplement 1. **eAppendix.** Derivation of Genetic Risk Equivalent (GRE) and Study Design and Study Population **eFigure.** Flowchart of Inclusion of Study Participants **eTable 1.** Information About Genotyping and Imputation **eTable 2.** Overview of Colorectal Cancer Related Single-Nucleotide Variations Identified in Genome-Wide Association Studies and Considered in this Analysis **eTable 3.** Distribution of BMI at Different Ages Among Cases With Colorectal Cancer and Controls **eTable 4.** Distribution of PRS and BMI Levels According to History of Colonoscopy **eTable 5.** Joint Associations of BMI and Polygenic Risk Score Levels With Colorectal Cancer **eTable 6.** Subgroup Analysis of Associations Between BMI and Colorectal Cancer
eReferences

Supplement 2.
Data Sharing Statement
