# Supplementary material for: Assessment of Body Mass Index, Polygenic Risk Score, and Development of Colorectal Cancer
Source: JAMA Netw Open. 2022 Dec 22;5(12):e2248447. doi: 10.1001/jamanetworkopen.2022.48447 (PMC9857417; doi:10.1001/jamanetworkopen.2022.48447)
Supplement: Supplement 1. — eAppendix. Derivation of Genetic Risk Equivalent (GRE) and Study Design and Study Population eFigure. Flowchart of Inclusion of Study Participants eTable 1. Information About Genotyping and Imputation eTable 2. Overview of Colorectal Cancer Related Single-Nucleotide Variations Identified in Genome-Wide Association Studies and Considered in this Analysis eTable 3. Distribution of BMI at Different Ages Among Cases With Colorectal Cancer and Controls eTable 4. Distribution of PRS and BMI Levels According to History of Colonoscopy eTable 5. Joint Associations of BMI and Polygenic Risk Score Levels With Colorectal Cancer eTable 6. Subgroup Analysis of Associations Between BMI and Colorectal Cancer eReferences [file jamanetwopen-e2248447-s001.pdf]

Supplemental Online Content

Chen X, Li H, Mandic M, Hoffmeister M, Brenner H. Assessment of body mass index, polygenic risk score, and development of colorectal cancer. *JAMA Netw Open*. 2022;5(12):e2248447. doi:10.1001/jamanetworkopen.2022.48447

eAppendix. Derivation of Genetic Risk Equivalent (GRE) and Study Design and Study Population
eFigure. Flowchart of Inclusion of Study Participants
eTable 1. Information About Genotyping and Imputation
eTable 2. Overview of Colorectal Cancer Related Single-Nucleotide Variations Identified in Genome-Wide Association Studies and Considered in this Analysis
eTable 3. Distribution of BMI at Different Ages Among Cases With Colorectal Cancer and Controls
eTable 4. Distribution of PRS and BMI Levels According to History of Colonoscopy
eTable 5. Joint Associations of BMI and Polygenic Risk Score Levels With Colorectal Cancer
eTable 6. Subgroup Analysis of Associations Between BMI and Colorectal Cancer
eReferences

This supplemental material has been provided by the authors to give readers additional information about their work.

© 2022 Chen X et al. *JAMA Network Open.*

eAppendix. Derivation of Genetic Risk Equivalent (GRE) and Study Design and Study Population

1. Derivation of genetic risk equivalent (GRE)

The concept of GRE was in analogy with the well-established concept of risk and rate advancement periods [1]. Briefly, consider an analysis based on a multivariable logistic regression:

$$\ln(\mathrm{R}) = a + b1 \cdot B + b2 \cdot P + \sum_{i=1}^{n} ci \cdot Fi$$

where ln(R) reflects the log odds of the disease risk, and a, b_1, b_2 and c_i (i = 1, . . ., n) refer to the intercept and model parameters for B [body mass index (BMI), categorized as 1 for the overweight or obese group and 0 for the group with normal weight], P (PRS percentile according to distribution of PRS among controls), and F (other covariates), respectively. The GRE was calculated as the ratio of b_1 and b_2, the estimated coefficients for BMI categories and the PRS from the regression models, and thus the properties of GRE follow from the properties of b_1 and b_2, which include consistency, asymptotic unbiasedness, and normality. Using the delta method [2], the asymptotic variance of GRE can be derived as:

$$\mathrm{var(GRE)} = \frac{1}{{b_2}^2}\left[\mathrm{var}(b_1) - 2\cdot\left(\frac{b_1}{b_2}\right)\cdot\mathrm{cov}(b_1, b_2) + \left(\frac{b_1}{b_2}\right)^2\cdot\mathrm{var}(b_2)\right]$$

As the GRE is asymptotically normal, its 95% confidence intervals can be easily calculated using the square root of var(GRE):

$$\mathrm{GRE} \pm 1.96\sqrt{\mathrm{var\ (GRE)}}$$

2. Study design and study population

The DACHS (Darmkrebs: Chancen der Verhütung durch Screening [German]) study is a large ongoing population-based case-control study initiated in 2003 and carried out in the Rhine-Neckar region in southwest Germany. Briefly, German-speaking patients who are aged ≥30 years (no upper age limit) and diagnosed with CRC for the first time are recruited from over 20 hospitals in the study region. Overall, the recruited patients represent about 50% of all eligible patients in this study area. Using frequency matching by age (5-year groups), sex, and county of residence, controls are randomly selected from population registries (participation rate: approximately 50%). Only controls without a history of CRC are included.

The current analysis includes cases and controls recruited in 2013-2017 with genetic and BMI data. Data on sociodemographic characteristics, lifestyles, medical and family history were collected in an approximately one-hour personal interview conducted by trained interviewers using a standardized questionnaire. Medical data were extracted for all cases from hospital charts. Interviews for patients were usually scheduled in hospital for cases, and were scheduled at homes for controls. In addition, blood and buccal swab samples were collected.

© 2022 Chen X et al. *JAMA Network Open.*

eFigure. Flowchart of Inclusion of Study Participants

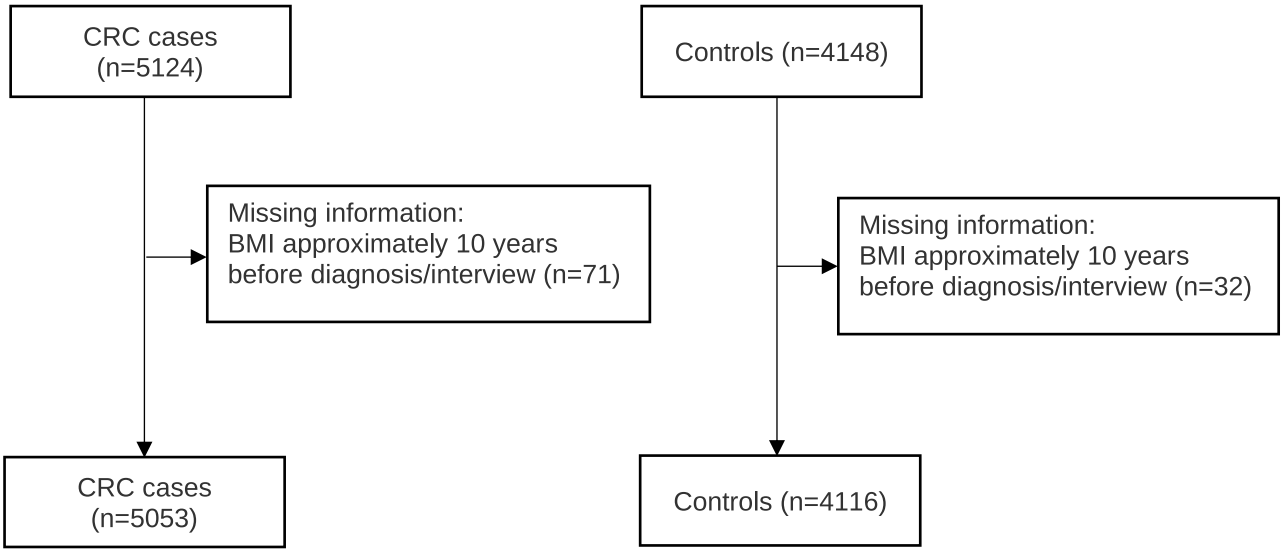

Abbreviations: BMI, body mass index; CRC, colorectal cancer.

© 2022 Chen X et al. *JAMA Network Open.*

eTable 1. Information About Genotyping and Imputation

Genotyping platform	CRC case (N)	Control (N)	Recruitment Period	Imputation
Illumina HumanCytoSNP	1681	1698	2003-2008	Haplotype Reference Consortium (version r1.1.2016)
Illumina HumanOmniExpress	660	489	2007-2010	
Illumina HumanOmniExpress	1173	624	2010-2015	
Illumina Infinium OncoArray	884	655	2003-2016	
Illumina Global Screening Array	655	650	2016-2017	

Note: We excluded triallelic SNPs, genotyped SNPs which had a low call rate (<98%), lack of Hardy-Weinberg equilibrium in control individuals ($p<1\times10^{-4}$), or low minor allele frequency (<0.1%), and those not assigned an rs number. More details can be found in the previous studies by Peters et al [3] and Schumacher et al [4].

© 2022 Chen X et al. *JAMA Network Open.*

eTable 2. Overview of Colorectal Cancer Related Single-Nucleotide Variations Identified in Genome-Wide Association Studies and Considered in this Analysis

SNV	Locus	Position	Gene	Risk allele	Beta
rs4360494	1p34.3	38455891	SF3A3	G	0.0379
rs12144319	1p32.3	55246035	TTC22	C	0.0661
rs72647484	1p36.12	22587728	/	T	0.0504
rs7542665	1p31.3	62673037	L1TD1	C	0.0334
rs6678517	1q25.3	183002639	LAMC1	A	0.073
rs17011141	1q41	222112634	LINC02257	G	0.0877
rs448513	2q24.2	159964552	TANC1	C	0.0054
rs11884596	2q33.1	199612407	LOC105373831	C	0.0342
rs983402	2q33.1	199781586	/	T	0.0622
rs7606562	2p16.3	48686695	PPP1R21	T	0.0414
rs11692435	2q11.2	98275354	ACTR1B	G	0.0492
rs3731861	2q35	219191256	/	T	0.0613
rs10049390	3q22.2	133701119	SLCO2A1	A	0.0455
rs13086367	3q13.2	112903888	/	A	0.0463
rs72942485	3q13.2	112999560	BOC	G	0.0545
rs9831861	3p21.1	53088285	/	G	0.0294
rs35470271	3p22.1	40915239	LOC105377043	G	0.0994
rs12635946	3q13.2	112916918	/	C	0.0334
rs113569514	3q22.2	133748789	SLCO2A1	T	0.0414
rs9876206	3q26.2	169517436	LRRC34	C	0.0453
rs6781752	3p14.1	66365163	SLC25A26	A	0.0597
rs11727676	4q31.21	145659064	HHIP	C	0.0093
rs1391441	4q24	106128760	/	A	0.0148
rs13149359	4q22.2	94938618	/	A	0.052
rs7708610	5p13.1	40102443	/	A	0.0384
rs78368589	5p15.33	1240204	SLC6A18	T	0.0786
rs145364999	5q21.1	98206082	CHD1	T	0.3496
rs2735940	5p15.33	1296486	TERT	G	0.0865
rs12514517	5p13.1	40280076	/	A	0.1013
rs755229494	5q22.2	112097351	APC	G	0.6286
rs12659017	5q23.2	125988175	/	G	0.0374
rs4976270	5q31.1	134467220	/	C	0.0693
rs13204733	6p12.1	55566108	/	G	0.0643
rs116685461	6p21.33	31315512	/	G	0.0655
rs9271695	6p21.32	32593080	/	G	0.0889
rs2516420	6p21.33	31449620	MICB-DT	C	0.1091
rs116353863	6p21.33	31010185	/	C	0.1202
rs16878812	6p21.31	35569562	/	A	0.0778
rs9470361	6p21.2	36623379	/	A	0.054
rs62404966	6p12.1	55712124	BMP5	C	0.0724
rs3131043	6p21.33	30758466	HCG20	G	0.0294

© 2022 Chen X et al. *JAMA Network Open.*

rs2070699	6p24.1	12292772	EDN1	T	0.0294
rs1476570	6p22.1	29809860	/	A	0.0492
rs3830041	6p21.32	32191339	NOTCH4	T	0.0645
rs6928864[a]	6q21	105966894	/	C	0.0531
rs62396735	6p21.1	41702582	/	C	0.033
rs12672022	7p13	45136423	/	T	0.0067
rs80077929	7p12.3	46094089	/	T	0.0093
rs10951878	7p12.3	46926695	/	C	0.0531
rs3801081	7p12.3	47511161	TNS3	G	0.0253
rs7013278	8q24.21	128414892	/	T	0.0091
rs4313119	8q24.21	128571855	/	G	0.0518
rs16892766	8q23.3	117630683	/	C	0.2099
rs6469654	8q23.3	117632965	/	G	0.0677
rs117079142	8q24.11	117790914	LOC112268030	A	0.1139
rs6983267	8q24.21	128413305	/	G	0.1052
rs34405347	9q22.33	101679752	/	T	0.0089
rs1537372	9p21.3	22103183	CDKN2B-AS1	G	0.012
rs10980628	9q31.3	113671403	LPAR1	C	0.0511
rs12217641	10p14	8663875	/	C	0.0069
rs10786560	10q24.2	101315166	/	G	0.0082
rs1250567	10q22.3	81046265	ZMIZ1	C	0.047
rs11255841	10p14	8739580	/	T	0.1064
rs10821907	10q11.23	52648454	/	C	0.073
rs704017	10q22.3	80819132	ZMIZ1-AS1	G	0.0765
rs11190164	10q24.2	101351704	/	G	0.0889
rs12246635	10q25.2	114288619	VTI1A	C	0.0975
rs11196170	10q25.2	114722621	TCF7L2	A	0.0527
rs7946853	11q13.4	74409077	CHRDL2	C	0.0119
rs55864876	11q22.1	100717136	ARHGAP42	G	0.015
rs2186607	11q22.1	101656397	/	T	0.0483
rs61389091	11q13.4	74427921	CHRDL2	C	0.1934
rs4450168	11p15.4	10286755	SBF2	C	0.0413
rs174533	11q12.2	61549025	MYRF	G	0.0636
rs7121958	11q13.4	74280012	/	G	0.078
rs3087967	11q23.1	111156836	C11orf53	T	0.1122
rs4759277	12q13.3	57533690	LRP1	A	0.0285
rs1427760	12q24.21	115100714	/	C	0.0424
rs3217874	12p13.32	4400808	CCND2	T	0.0453
rs10849433	12p13.31	6406904	LOC105369624	C	0.0468
rs11610543	12q12	43134191	/	G	0.0474
rs35808169	12p13.32	4368607	CCND2-AS1	C	0.089
rs3217810	12p13.32	4388271	CCND2	T	0.1181
rs2250430	12p13.31	6421174	PLEKHG6	T	0.0597
rs77969132	12p11.21	31594813	DENND5B	T	0.1583
rs12372718	12q13.12	51171090	ATF1	G	0.0896

© 2022 Chen X et al. *JAMA Network Open.*

rs597808	12q24.12	111973358	ATXN2	G	0.0737
rs7300312	12q24.21	115890922	LOC105370003	C	0.066
rs2710310	12p13.2	12035649	ETV6	C	0.0145
rs78341008	13q22.1	73791554	/	C	0.0109
rs8000189	13q34	111075881	COL4A2	T	0.0473
rs45597035	13q22.1	73649152	KLF5	A	0.0495
rs1924816	13q22.1	73997961	/	A	0.0506
rs7333607	13q13.3	37462010	SMAD9	G	0.0758
rs1330889	13q22.3	78609615	LINC00446	C	0.0453
rs377429877	13q13.2	34092164	STARD13	C	0.0468
rs1951864	14q22.2	54369299	LOC107984676	A	0.0059
rs17094983	14q23.1	59189361	/	G	0.0062
rs8020436	14q23.1	59208437	/	A	0.0294
rs35107139	14q22.2	54419106	BMP4	C	0.0912
rs4901473	14q22.2	54445157	/	G	0.0465
rs745213	15q23	68060389	MAP2K5	G	0.0072
rs12594720	15q22.31	67007018	SMAD6	C	0.0246
rs56324967	15q22.33	67402824	SMAD3	C	0.0689
rs17816465	15q13.3	33156386	FMN1	A	0.069
rs12708491	15q13.3	32992836	/	G	0.0464
rs2293581	15q13.3	33010736	/	A	0.1248
rs7495132	15q26.1	91172901	/	T	0.0453
rs9930005	16q23.2	80043258	/	C	0.0061
rs12447408	16q24.1	86252544	/	A	0.0079
rs9924886	16q22.1	68743939	CDH3	A	0.055
rs12149163	16q24.1	86339315	/	T	0.0487
rs62042090	16q24.1	86703949	/	T	0.0481
rs983318	17q24.3	70413253	LINC00673	A	0.0397
rs73975586	17p13.3	814243	/	A	0.0497
rs1078643	17p12	10707241	/	A	0.0747
rs75954926	17q25.3	81061048	LOC107987251	G	0.0882
rs373585858	17q25.3	80394556	HEXD	A	0.1103
rs4968127	17p13.3	809643	NXN	G	0.0514
rs11874392	18q21.1	46453156	SMAD7	A	0.1606
rs73068325	19q13.43	59079096	/	T	0.0066
rs34797592	19p13.11	16417198	/	T	0.0824
rs28840750	19q13.11	33519927	RHPN2	T	0.1939
rs1963413	19q13.2	41871573	/	A	0.0441
rs12979278	19q13.33	49218602	MAMSTR	T	0.0293
rs2738783	20q13.33	62308612	/	T	0.006
rs6067417	20q13.13	48983697	LOC105372657	C	0.0331
rs6031311	20q13.12	42666475	TOX2	T	0.0362
rs6091189	20q13.13	49256285	RIPOR3	T	0.0549
rs994308	20p12.3	6603622	/	C	0.0626
rs28488	20p12.3	6762221	/	T	0.0714

rs556532366	20p12.3	8568071	PLCB1	T	0.0715
rs189583	20p12.3	6376457	/	G	0.0795
rs4813802	20p12.3	6699595	/	G	0.0819
rs11087784	20p12.3	7740976	/	G	0.0874
rs6066825	20q13.13	47340117	PREX1	A	0.0719
rs6063514	20q13.13	49055318	/	C	0.0547
rs13831	20q13.32	57475191	GNAS	G	0.0334
rs1741640	20q13.33	60932414	LAMA5	C	0.1146
rs6058093	20q11.22	33213196	PIGU	C	0.045

"/": SNVs are not located in gene region.
[a]For building the PRS, the missing reference SNV was replaced by rs6904092 (linkage disequilibrium, D'=1 and r^2=1).
Abbreviations: A, adenine; C, cytosine; G, guanine; OR, odds ratio; T, thymine; SNV, single-nucleotide variation.

© 2022 Chen X et al. *JAMA Network Open.*

eTable 3. Distribution of BMI at Different Ages Among Cases With Colorectal Cancer and Controls

**BMI at Age (years)**	**BMI (kg/m^2)**	**CRC case, N (%)**	**Control, N (%)**	***P* value**
20	Total	4921	4028	
	<18.5	272 (5.5)	272 (6.8)	
	18.5- <25	3708 (75.4)	3178 (78.9)	
	25-<30	804 (16.3)	522 (13.0)	<0.001
	≥30	137 (2.8)	56 (1.4)	
30	Total	4908	4019	
	<18.5	95 (1.9)	108 (2.7)	
	18.5- <25	3172 (64.6)	2811 (69.9)	
	25-<30	1368 (27.9)	962 (23.9)	<0.001
	≥30	273 (5.6)	138 (3.4)	
40	Total	4886	4020	
	<18.5	44 (0.9)	57 (1.4)	
	18.5- <25	2397 (49.1)	2295 (57.1)	
	25-<30	1938 (39.7)	1385 (34.5)	<0.001
	≥30	507 (10.4)	283 (7.0)	
50	Total	4714	3890	
	<18.5	26 (0.6)	32 (0.8)	
	18.5- <25	1731 (36.7)	1741 (44.8)	
	25-<30	2161 (45.8)	1675 (43.1)	<0.001
	≥30	796 (16.9)	442 (11.4)	
60	Total	3983	3309	
	<18.5	23 (0.6)	15 (0.5)	
	18.5- <25	1115 (28.0)	1209 (36.5)	
	25-<30	1947 (48.9)	1566 (47.3)	<0.001
	≥30	898 (22.5)	519 (15.7)	
70	Total	2486	2040	
	<18.5	16 (0.6)	10 (0.5)	
	18.5- <25	641 (25.8)	641 (31.4)	
	25-<30	1225 (49.3)	1037 (50.8)	<0.001
	≥30	604 (24.3)	352 (17.3)	
80	Total	794	662	
	<18.5	10 (1.3)	7 (1.1)	
	18.5- <25	229 (28.8)	228 (34.4)	
	25-<30	412 (51.9)	320 (48.3)	0.15
	≥30	143 (18.0)	107 (16.2)	
Approximately 10 years before diagnosis/interview	Total	5053	4116	
	<18.5	39 (0.8)	30 (0.7)	
	18.5- <25	1502 (29.7)	1550 (37.7)	
	25-<30	2372 (46.9)	1885 (45.8)	<0.001
	≥30	1140 (22.6)	651 (15.8)	

Abbreviations: BMI, body mass index; CRC, colorectal cancer.

© 2022 Chen X et al. *JAMA Network Open.*

eTable 4. Distribution of PRS and BMI Levels According to History of Colonoscopy

PRS and BMI	Cases			P^b	Controls			P^b
	History of colonoscopy				History of colonoscopy			
	No (N=3718)	Yes (N=1316)			No (N=1636)	Yes (N=2471)		
		<50 years[a]	≥50 years[a]			<50 years[a]	≥50 years[a]	
PRS, N (%)				0.83				0.56
Low	775 (20.8)	60 (20.2)	220 (21.6)		565 (34.5)	120 (30.2)	687 (33.1)	
Medium	1181 (31.8)	92 (31.0)	335 (32.9)		533 (32.6)	139 (34.9)	697 (33.6)	
High	1762 (47.4)	145 (48.8)	464 (45.5)		538 (32.9)	139 (34.9)	689 (33.2)	
BMI[c] (kg/m^2), N (%)				0.35				<0.001
<25	1158 (31.1)	89 (30.0)	289 (28.4)		701 (42.8)	164 (41.2)	713 (34.4)	
25-<30	1736 (46.7)	145 (48.8)	481 (47.2)		704 (43.0)	167 (42.0)	1010 (48.7)	
≥30	824 (22.2)	63 (21.2)	249 (24.4)		231 (14.1)	67 (16.8)	350 (16.9)	

[a]Age when first colonoscopy was conducted. 28 participants had missing information on year of colonoscopy examinations.
[b]Results were compared using Chi-square tests.
[c]BMI about 10 years before diagnosis/interview.
Abbreviations: BMI, body mass index; PRS, polygenic risk score.

© 2022 Chen X et al. *JAMA Network Open.*

eTable 5. Joint Associations of BMI and Polygenic Risk Score Levels With Colorectal Cancer

**BMI (kg/m^2, about 10 years before diagnosis/interview)**	**OR (95% CI)[a]**		
	**Low PRS[b]**	**Medium PRS[b]**	**High PRS[b]**
<25	Ref.	1.67 (1.36, 2.04)	2.41 (1.98, 2.93)
25-<30	1.35 (1.10, 1.65)	2.12 (1.75, 2.57)	3.14 (2.60, 3.79)
≥30	1.97 (1.53, 2.56)	2.79 (2.19, 3.56)	3.82 (3.03, 4.82)

[a]Ajusted for age, sex, education, smoking status, alcohol consumption, physical activity, red and processed meat intake, history of colonoscopy, history of diabetes, family history of colorectal cancer, use of statins, and use of nonsteroidal anti-inflammatory drugs.
[b]PRS was categorized according to tertiles of PRS (low, medium, and high levels) among controls.
Abbreviations: BMI, body mass index; CI, confidence interval; OR, odds ratio; PRS, polygenic risk score; Ref., reference.

© 2022 Chen X et al. *JAMA Network Open.*

eTable 6. Subgroup Analysis of Associations Between BMI and Colorectal Cancer

**Subgroup**		**BMI about 10 years before diagnosis/interview**					***P* value**[b]
		**Category (kg/m²)**	**CRC case, N (%)**	**Control, N (%)**	**OR (95% CI)**[a]	**GRE (95% CI)**	
Age (year)	≤55	<25	296 (46.0)	270 (56.1)	Ref.	Ref.	0.33
		25-<30	236 (36.7)	154 (32.0)	1.43 (1.07, 1.92)	29.3 (3.1, 55.4)	
		≥30	111 (17.3)	57 (11.9)	1.43 (0.96, 2.14)	29.3 (-5.3, 63.8)	
	>55	<25	1205 (28.0)	1283 (36.0)	Ref.	Ref.	
		25-<30	2087 (48.6)	1705 (47.8)	1.29 (1.15, 1.45)	19.4 (10.2, 28.6)	
		≥30	1004 (23.4)	579 (16.2)	1.73 (1.49, 2.00)	41.8 (29.2, 54.4)	
Sex	Female	<25	783 (40.4)	765 (49.1)	Ref.	Ref.	0.78
		25-<30	741 (38.2)	562 (36.0)	1.29 (1.09, 1.52)	19.4 (6.1, 32.8)	
		≥30	416 (21.4)	232 (14.9)	1.59 (1.28, 1.98)	35.4 (17.3, 53.5)	
	Male	<25	718 (23.9)	788 (31.7)	Ref.	Ref.	
		25-<30	1582 (52.8)	1297 (52.1)	1.31 (1.14, 1.51)	20.6 (9.4, 31.9)	
		≥30	699 (23.3)	404 (16.2)	1.77 (1.48, 2.13)	43.6 (28.1, 59.0)	
History of colonoscopy	No	<25	1128 (31.0)	693 (42.9)	Ref.	Ref.	0.18
		25-<30	1699 (46.8)	694 (43.0)	1.36 (1.18, 1.57)	22.0 (11.4, 32.6)	
		≥30	807 (22.2)	227 (14.1)	1.86 (1.54, 2.25)	44.4 (29.3, 59.5)	
	Yes	<25	373 (28.6)	860 (35.3)	Ref.	Ref.	
		25-<30	624 (47.8)	1165 (47.9)	1.22 (1.03, 1.44)	17.5 (2.5, 32.6)	
		≥30	308 (23.6)	409 (16.8)	1.53 (1.24, 1.88)	37.5 (17.5, 57.5)	
Family history of colorectal cancer	No	<25	1317 (31.2)	1381 (38.3)	Ref.	Ref.	0.15
		25-<30	1966 (46.6)	1664 (46.2)	1.25 (1.11, 1.40)	18.3 (8.6, 27.9)	
		≥30	934 (22.1)	560 (15.5)	1.66 (1.43, 1.92)	41.5 (28.0, 55.0)	
	Yes	<25	184 (25.5)	172 (38.8)	Ref.	Ref.	
		25-<30	357 (49.4)	195 (44.0)	1.94 (1.41, 2.69)	38.1 (16.8, 59.4)	
		≥30	181 (25.1)	76 (17.2)	2.22 (1.49, 3.33)	45.8 (20.0, 71.7)	

[a]Adjusted for age, sex, education, smoking status, alcohol consumption, physical activity, red and processed meat intake, history of colonoscopy, history of diabetes, family history of colorectal cancer, use of statins, use of nonsteroidal anti-inflammatory drugs, and the polygenic risk score (per 10 percentiles, continuous variable) but without the pertinent stratification factors (age/sex/history of colonoscopy/family history of colorectal cancer).

[b]Interactions were tested by including a cross-product term of BMI (categorical variable) and pertinent stratification factors (categorical variable) in the regression models.

Abbreviations: BMI, body mass index; CI, confidence interval; CRC, colorectal cancer; GRE, genetic risk equivalent; OR, odds ratio; Ref., reference.

© 2022 Chen X et al. *JAMA Network Open.*

eReferences

1. Brenner H, Gefeller O, Greenland S. Risk and rate advancement periods as measures of exposure impact on the occurrence of chronic diseases. *Epidemiology*. 1993;4(3):229-236. doi:10.1097/00001648-199305000-00006
2. Bishop YMM, Fienberg SE, Holland PW. *Discrete Multivariate Analysis: Theory and Practice.* MIT press; 1975.
3. Peters U, Jiao S, Schumacher FR, et al. Identification of Genetic Susceptibility Loci for Colorectal Tumors in a Genome-Wide Meta-analysis. *Gastroenterology*. 2013;144(4):799-807.e24. doi:10.1053/j.gastro.2012.12.020
4. Schumacher FR, Schmit SL, Jiao S, et al. Genome-wide association study of colorectal cancer identifies six new susceptibility loci. *Nat Commun*. 2015;6(1):7138. doi:10.1038/ncomms8138

© 2022 Chen X et al. *JAMA Network Open.*
