# Supplementary material for: Assessment of Body Mass Index, Polygenic Risk Score, and Development of Colorectal Cancer
Source: JAMA Netw Open. 2022 Dec 22;5(12):e2248447. doi: 10.1001/jamanetworkopen.2022.48447 (PMC9857417; doi:10.1001/jamanetworkopen.2022.48447)
Supplement: Supplement 2. — Data Sharing Statement [file jamanetwopen-e2248447-s002.pdf]

Data Sharing Statement

Chen. Assessment of Body Mass Index, Polygenic Risk Score, and Development of Colorectal Cancer. *JAMA Netw Open*. Published December 22, 2022.
doi:10.1001/jamanetworkopen.2022.48447

Data
Data available: No
